# An extension of the extended parallel process model to promote heart-healthy exercise behavior: An experimental study

**DOI:** 10.34172/hpp.2022.47

**Published:** 2022-12-31

**Authors:** Rashmi Thapaliya, Glenn Leshner, Pragya Sharma Ghimire, Amir Bhochhibhoya

**Affiliations:** ^1^School of Communication and Journalism, Eastern Illinois University, IL, USA; ^2^Gaylord College of Journalism & Mass Communication, University of Oklahoma, Oklahoma, USA; ^3^School of Health and Human Performance, Kean University, New Jersey, USA

**Keywords:** Exercise, Health Belief Model, Heart diseases, Self-efficacy

## Abstract

**Background:** The prevalence of heart disease has increased and is a leading cause of death in the U.S. Despite the importance of physical activity, only one-third of adults in the United States meet the amount of physical activity recommended by the Centers for Disease Control and Prevention (CDC). The purpose of this study was to extend the extended parallel process model (EPPM) by adding a ‘barrier’ (a construct from Health Belief Model) and exploring the roles of threat, efficacy, and barrier on participants’ self-efficacy, attitudes, and intentions toward exercise.

**Methods:** A between-subject experimental design was conducted online in 2018 in the U.S. A total of 446 participants were recruited from the Amazon Mechanical Turk age 18 or above. The participants were first provided with stimuli messages about physical activity behaviors. Then participants’ responses to self-efficacy, intention, and attitudes toward exercise were assessed.

**Results:** The results found an interaction between efficacy and barrier to participants’ attitudes toward exercise [F(1,435)=4.35, *P*=0.038, η^2^_part_=0.01]. The results also showed that there was a statistically significant effect of barriers on participants’ self-efficacy regarding exercise behavior [F(1,442)=4.21, *P*=0.04, η^2^_part_=0.009]. However, three-way interactions of threat, efficacy, and barrier were not found in attitudes or intentions to exercise.

**Conclusion:** The findings suggested that addressing an individual’s perceived barrier regarding a health behavior may lead to an increase in self-confidence ensuing in higher physical activity. Future studies should further explore how addressing barriers may influence other health behaviors to design unique and effective health messages.

## Introduction

 Heart disease, which encompasses several types of heart conditions, is a major public health problem. Despite the advancements in technologies and treatments, the prevalence of heart disease has increased and is a leading cause of death for both men and women in the United States.^1^ Coronary artery disease (CAD) - a chronic condition that reduces blood flow to the heart - is the most common heart disease in the United States. Based on the reports from the American Heart Association (AHA), 382 820 people died of CAD in the United States in 2020.^2,3^ The CAD is also a major cause of disability. Currently, CAD is prevalent among more than 6.7% of adults aged 20 and older in the United States.^4^

 Engaging in daily physical activity is a major step to reduce the risk of CAD as well as several other chronic conditions such as obesity, high cholesterol, and high blood pressure, which further exacerbate CAD.^5^ Despite its importance, only one in three adults meets the amount of physical activity recommended by the Centers for Disease Control and Prevention (CDC) (United States Department of Health and Human Services).^6^ This portrays a significant lack of physical activity in lifestyle and warrants a motivating factor to enhance physical activity.

 Past studies have suggested that studies utilizing a strong theoretical basis were more effective for behavior change application than those with no theoretical underpinnings.^7^ Among the theories used by scholars, the Health Belief Model (HBM) and the extended parallel process model (EPPM) are commonly used fear appeal theories.

 Fear appeal messages have been conceptualized as messages that “directly associate the targeted behavior (e.g., tobacco use) with a threat (e.g., disease, death)”.^8^ It may include gruesome content such as pictures of a severely damaged lung or the use of vivid language such as “thick purulent, choking secretions welled into the tracheotomy wound” as well as measured perceived fear by the participants.^9,10^

 The EPPM describes the process of what happens when an individual is exposed to a fear appeal including the components of threat and efficacy. According to EPPM, when an individual is exposed to a fear appeal then two appraisals are initiated.^10,11^ If the first appraisal suggests the threat is moderate to high, then it stimulates fear that motivates the second appraisal.^10^ The second appraisal assesses the efficacy of the recommended response. Efficacy is an external stimulus that exists as an environmental or message cue.^12^ Efficacy includes response efficacy and self-efficacy. Perceived response efficacy is an individual’s belief in the effectiveness of the recommended response whereas perceived self-efficacy is an individual’s belief in their ability to perform the recommended response. When the individual perceives the threat is low, then there is no motivation to further process the message. When the individual perceives that the threat is high and the actions recommended in the message are effective, and they can perform the actions, then the danger control process is initiated. In the danger control process, individuals are motivated to protect themselves through message acceptance responses such as attitude, intention, or behavior change to avoid negative consequences.^11^ On the other hand, when the threat is high but perceived efficacy is low, then it intensifies the fear and leads to ineffective defensive mechanisms such as ignoring the message or denying that the threat exists.^10^ Also, individual differences such as previous experience, culture, personality, and personal characteristics influence how people will perceive a threat message.^10^

 The original HBM posits that the health message will bring optimal behavior change if successfully targeted perceived: susceptibility, severity, benefits, and barriers. According to the HBM, the message should communicate an underlying threat (high susceptibility and high severity) to health as well as convey the perceived benefits of the action (change in the behavior). To convince an individual to follow the recommended action, the perceived benefits should outweigh the perceived barriers.^12^

**Figure 1:**
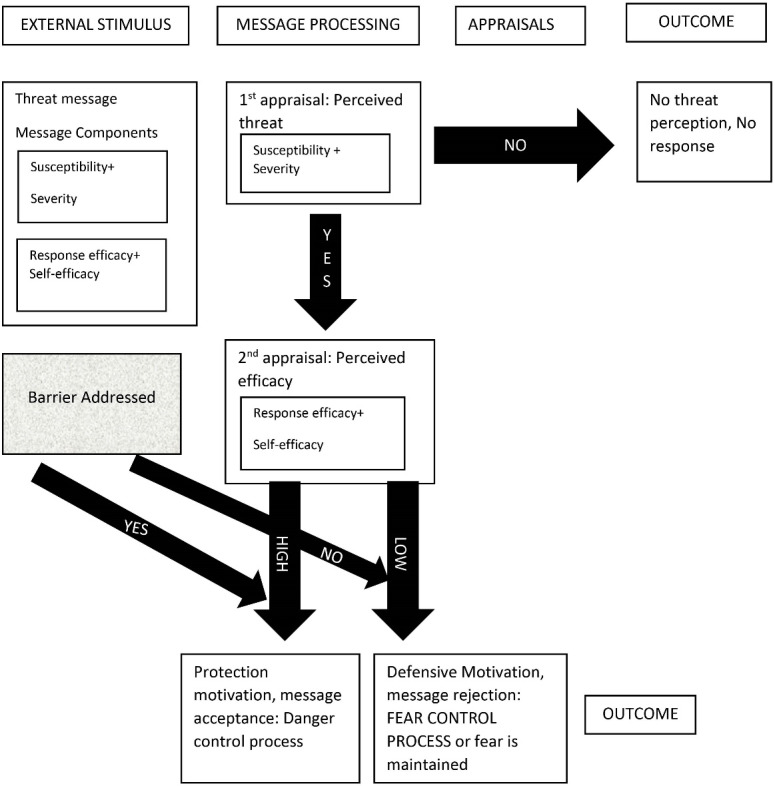
Integrated EPPM model with barrier added

 Both EPPM and HBM have grown largely out of the social psychological literature and both utilize the fear of the negative consequence of a behavior as an important motivator of behavior change.^13^ Researchers in the past have advocated for integrating theories to help the field of health communication advance.^14,15^ Noar suggests that it can be beneficial to include multiple theories for a prevention effort, especially if the two theories complement each other.^13^ The key constructs of EPPM and HBM match each other (susceptibility, severity, and self-efficacy) but EPPM does not include the construct of perceived barriers.^16,17^ Perceived barriers include economic as well as other costs related to adopting recommended actions.^18^ A comprehensive review of HBM found that the perceived barrier was the most powerful of the HBM dimensions across various study designs and behaviors.^19^ Thus, researchers have suggested that a perceived barrier should be added as a variable to EPPM to increase its explanatory and predictive power.^20^ Researchers have extended the EPPM in the past by adding different cognitive and emotional elements to make the model stronger but extending EPPM by adding the ‘barrier’ construct from HBM while designing a message to promote exercise is the first of its kind.^21,22^ See Figure 1 for the proposed integrated model that adds the variable barrier to the EPPM model.

 Several past studies have recommended that to increase physical activity among an inactive population, health interventions should be focused on addressing the perceived barriers.^23-25^ Researchers have concluded that when perceived barriers are recognized and addressed in the intervention programs, it helps to increase the self-efficacy of individuals to promote physical activities for healthy living.^26-28^

 The purpose of this study is to explore if adding a barrier to the EPPM model will add to its utility to promote physical activity. In the context of heart health, previous studies have suggested that people of various ages and patients have cited a lack of ‘time’ as a key barrier to being physically active.^23,24,29-31^ Thus, this study is designed to extend the EPPM model by addressing the barrier, ‘time’ through narrative messages to positively influence participants’ behavior to exercise regularly.

## Materials and Methods

###  Participants and study design

 The study utilized a between-subject experimental design, and the participants were randomly assigned to one of the eight experimental groups. The groups were based on 2 threats (high vs. low) × 2 efficacy (high vs. low) × 2 barriers (addressed vs. not addressed) between-subjects design. The study was conducted in 2018. A total of 446 participants were recruited online utilizing Amazon Mechanical Turk (MTurk) at a 95% HIT (Human Intelligence Task) approval rate. The percentage signifies online investigations submitted by the users (workers) that have been approved by the requesters to ensure response quality. The eligibility criteria for the participants were Amazon MTurk users (workers) who were 18 or above. Scholars have noted that Amazon MTurk can be used as a good alternative to collecting data from other sources such as students, professionals, and online professional panels.^32^

###  Procedure

 Before the participants started the online experiment, informed consent was received. After consent, the participants were randomly assigned to one of the eight-threat × efficacy × barrier conditions. In the beginning, the participants were asked to answer questions about their exercise behaviors. The participants were then asked to read a narrative about exercising for heart health. After reviewing the narrative, participants were presented with a questionnaire regarding the exposed message.

###  Manipulation 

 The variables that were manipulated in the study threat, efficacy, and barriers were integrated into the narratives. To confirm the manipulations: threat (high vs. low), efficacy (high vs. low), and barrier (addressed vs. not addressed), a pilot test was conducted. For the manipulation check, 50 participants (28 males, 22 females) were recruited online using the Amazon MTurk system. A repeated measure design was implemented where participants were asked to view two messages for exercise behaviors. The first message contained a narrative with high threat, high efficacy, and barrier addressed. The second message included a message that contained the low threat, low efficacy, and no barrier addressed. After viewing each message, the participants were asked to answer questions about the content of the message. A set of paired-sample t-tests were conducted to check the message content manipulation for the exercise message. The results showed that the manipulation of the content of threat, efficacy, and barrier in the messages was successful.

 After the manipulation check, one narrative message stimulus was devised for each condition. Research studies conducted to influence health behaviors that compared narrative and non-narrative messages have found that narratives are better at changing knowledge, attitudes, and behavioral intentions than non-narrative messages.^33^ Thus, the study used narratives as the stimuli messages. For high-threat messages, some severe effects of not exercising on heart health were comprehended such as “*If I kept being sedentary, there will be severe health risks including high blood pressure, heart attack, and other heart-related problems that can even cause death*.” Low-threat messages, in contrast, described some less severe effects of not exercising such as “*If I kept being sedentary, there will be some health risks including low energy, high stress, bad mood, and heart disease*.” Similarly, for the high efficacy message, response efficacy was maximized by the narrator’s emphasis on the effectiveness of brisk walking to improve heart health. The self-efficacy was increased by citing the ease of taking a walk and the narrator suggesting initiating exercising with moderate physical exercises. For the low efficacy message, response efficacy was minimized by suggesting that walking regularly was not very effective in improving heart health. The self-efficacy was minimized by the low confidence of the narrator in their ability to exercise regularly. The barrier was manipulated by addressing one of the most common barriers to the exercise, “*time.*” The message that addressed the barrier of time included, “*When I feel like I will not have enough time to exercise, I break up my exercise into smaller chunks of time during the day. It’s all about what works best for me, as long as I am doing physical activity at moderate or vigorous effort for at least 10 minutes at a time.*” The second message condition for the independent variable barrier was manipulated by not addressing the barrier at all.

 The stimulus layout, colors, size, and typeface of each message was held constant across the eight conditions. The changes in the text reflected the accurate information retrieved from the CDC and the AHA websites. For the messages, Riley was chosen as the narrator’s name because “Riley” was listed as the top 20 gender-neutral names in the United States and a gender-neutral name was chosen to control for the effect of the narrators’ genders on participants’ perception of the messages.^34^

###  Measurement

 After reading the narrative, the participants’ responses to self-efficacy, intention, and attitudes toward exercise were assessed. To assess self-efficacy, intention, and attitude towards exercise questionnaire from Richards and Johnson’s study, which reported adequate reliability, was utilized in the current study.^35^ The perceived self-efficacy was measured using six items such as “*Exercising for 30 minutes per day 5 days a week during the next month will be easy for me*” (Cronbach α = 0.924). Similarly, the intentions were measured using a two-items such as “*I intend to exercise for at least 30 min per day*” (Cronbach α = 0.960). The participants’ attitudes towards exercise were measured using a four-item semantic differential scale. The statement asked the participants to indicate the extent to which engaging in exercise during the next month would be good/bad, enjoyable/not enjoyable, unwise/wise, and beneficial/not beneficial. The participants were also asked to rate their exercise behaviors: “*how often do you participate in following physical activities in a typical week?”* on a 7-point scale where “*1*” = “*not at all*” and “*7*” = “*very much*” (Cronbach α = 0.865).

###  Statistical analysis

 Descriptive statistics were used to report the demographic characteristics and exercise behavior. The correlation matrix and Cronbach Alpha were used to assess scale reliability. One-way analysis of variance (ANOVA) was used to determine the mean difference between the participants’ conditions in different groups. Analysis of covariance (ANCOVA) was used to test the main effects and interactions between independent variables. Participant’s exercise behavior was used as a covariate. All data were analyzed using SPSS (Version 24.0, Armonk, NY). The alpha level (α) was set at 0.05 to determine statistical significance.

## Results

 A majority of the participants were female (247, 55.4%), white or Caucasian (327, 73.3%), with the educational attainment of bachelor’s degrees (204, 45.7%), and full-time employed (317, 71.1%). Participants’ ages ranged from 19-76 years (M = 39.80, SD*=*12.420). The participants’ mean for the exercise behavior, was 2.89 (SD = 1.43), which shows that on average the participants were not very active physically. The number of participants in each group ranged from 51-65 (M = 39.80, SD = 12.420). Details on the demographic variables are presented in Table 1.

**Table 1:** Demographic characteristics (frequency and descriptive statistics)

**Characteristic**	**Number (%)**	**Total**
Gender		446
Male	198 (4.4)	
Female	247 (55.4)	
Other	1 (0.2)	
Race/Ethnicity		446
White or Caucasian	327 (73.3)	
Black or African American	36 (8.07)	
Hispanic	27 (6.1)	
Asian	40 (9)	
Native American	4 (0.9)	
Other	11 (2.5)	
Income		446
Less than $10 000	11 (2.5)	
$10 000-$49 999	187 (41.92)	
$50 000-$99 999	234 (52.45)	
$100 000 and above	14 (3.14)	
Education		446
Less than high school	4 (0.9)	
High school degree/GED	38 (8.5)	
Some college	123 (27.58)	
Bachelor’s degree and above	268 (60)	
Employment		
Full time	317 (71.1)	
Part-time	53 (11.9)	
Not employed	47 (10.5)	
Retired	28 (6.3)	
Marital status		446
Single (never been married)	173 (38.8)	
Married	216 (48.4)	
Widowed	9 (2)	
Separated	4 (0.9)	
Divorced	44 (9.9)	
Heart disease		446
Diagnosed	23 (5.2)	
Not diagnosed	419 (98.4)	
A close family member diagnosed	254 (57)	
Close family member not diagnosed	191 (42.8)	

 A one-way ANOVA found that there was no statistically significant difference between the participants’ ages and their random assignment to the messages with manipulation of threat; *F*(1,438) = 0.076, *P* = 0.78; efficacy, *F*(1,438) = 0.07, *P* = 0.97; and barrier, *F*(1,438) = 0.12, *P* = 0.72. Furthermore, there was no significant interaction of threat and barrier; *F*(1,438) = 0.34, *P* = 0.56; threat and efficacy; *F*(1,478) = 1.80, *P* = 0.18; barrier and efficacy *F*(1,438) = 0.05, *P* = 0.82, and threat, efficacy, and barrier *F*(1,438) = 1.18, *P* = 0.23. Therefore, the participants were randomly assigned to the different conditions of threat, efficacy, and barrier despite their ages. The Cronbach alpha for all variables was reported higher than 0.70, suggesting the instrument holds internal consistency for scale reliability.

 The study hypothesized that participants who read a message that addresses the barrier of the amount of time required to exercise will report higher perceived self-efficacy than the participants who read a message that does not address the barrier. The main effect of the barrier which addressed (or not addressed) on participants’ perceived self-efficacy about exercising (*F*(1,442) = 4.21, *P* = 0.04, *η*^2^_part _= 0.009) was statistically significant. Results further revealed that the participants in the barrier-addressed message condition reported a higher perceived self-efficacy about exercising (M = 4.88, SD = 1.46) than the participants in the barrier not addressed message condition (M = 4.54, SD = 1.56).

 The study also evaluated if a high-threat message that addresses the barrier, and amount of time required to exercise will report higher perceived self-efficacy, more positive attitudes towards exercising, and greater intentions to exercise than the participants who read a high-threat message that does not address the barrier to exercise. The interaction between threat and barrier on participants’ perceived self-efficacy on exercise behavior [*F*(1,436) = 0.88, *P* = 0.35, *η*^2^_part_ = .002), attitudes towards exercise [*F*(1,435) = 1.46, *P* = 0.23, *η*^2^_part_ = 0.003] and intentions to exercise [*F*(1,436) = 1.09, *P* = 0.29, *η*^2^_part_ = 0.002] were not significant.

 The study also assessed if a high threat and high efficacy message that addresses the barrier to exercise will report the most positive attitudes and the highest intentions to exercise than the participants in other message conditions. The three-way interaction of threat, efficacy, and barrier on: attitudes towards exercise [*F*(1,435) = 0.09, *P* = 0.76, *η*^2^_part_ = 0.000] and intentions to exercise [*F*(1,436) = 0.08, *P* = 0.77, *η*^2^_part_ = 0.000] were not significant.

**Figure 2:**
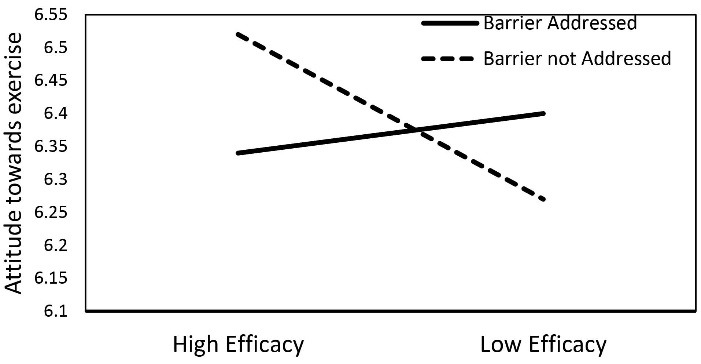
Interaction between efficacy and barrier on attitude towards exercise

 Results further showed that there was a significant two-way interaction between barrier and efficacy on the participants’ attitudes towards exercise [*F*(1,435) = 4.35, *P* = 0.038, *η*^2^_part_ = 0.01]. Also, participants in the high efficacy and barrier not addressed message conditions had more positive attitudes towards exercise (M = 6.53, SD = 0.62) than the participants in the high efficacy and barrier addressed message conditions (M = 6.35, SD = 0.88). Results further revealed that participants in the low efficacy and barrier addressed message conditions had a more positive attitude towards exercise (M = 6.40, SD = 0.88) than the participants in the low efficacy and barrier not addressed message conditions (M = 6.27, SD = 0.96). See Figure 2 for the interaction between efficacy and barrier on attitudes towards exercise. See Table 2 for a descriptive summary of the two-way interaction between barrier and efficacy on participants’ attitudes towards exercise.

**Table 2:** Descriptive summary for attitudes towards exercise (barrier x efficacy)

**Barrier**	**Efficacy**	**Mean**	**SD**
Addressed	High	6.35	0.88
	Low	6.40	0.88
Not addressed	High	6.53	0.62
	Low	6.27	0.96

 Results also showed that the main effect of the barrier on intentions to exercise [*F*(1,436) = 3.06, *P* = 0.084., *η*^2^_part_ = 0.007] was approaching significance. Results further revealed that participants in barrier-addressed message conditions reported more positive attitudes towards exercise (M = 5.01, SD = 1.66) than participants in barrier not addressed message conditions (M = 4.69, SD = 1.75).

## Discussion

 In the past, the EPPM has been used as a framework in research studies of various health issues such as cardiovascular diseases, hygienic behavior, public health emergency response, teen pregnancy, smoking, vaccination, and HIV/AIDS, among others.^36-40^ Similarly, the HBM has been utilized by health scholars to understand and predict different health behaviors ranging from a healthy diet, influenza vaccination, breast self-examination, mammography screening, oral hygiene, and weight management interventions among others.^41-46^ However, there have been few studies that have integrated multiple theories to increase those theories’ predictive power. Researchers have integrated the Theory of Planned Behavior (TPB) and EPPM to increase the explanatory power of TPB to predict intentions to exercise.^35^ The authors suggested that the integration of the two models of TPB and EPPM was better but still inadequate to predict exercise intentions and other psychological constructs may need to be considered for better predictions of exercise intentions.^35^ In tandem, Carcioppolo suggested adding a barrier to EPPM to increase its explanatory and predictive power.^20^ Thus, this study was designed to explore if adding a barrier to the EPPM model through narrative messages better influences participants’ attitudes and intentions to regularly exercise.

 The current study explored whether participants exposed to the barrier-addressed message condition would report higher perceived self-efficacy than the participants exposed to barrier not addressed message conditions. A statistically significant effect of barrier-addressed participants’ perceived self-efficacy was found for exercise behavior. The literature on HBM and EPPM has suggested that the perceived barrier must be addressed to help increase the self-efficacy of individuals regarding health behaviors.^26^ The current study showed that addressing barriers can increase the perceived self-efficacy of individuals for exercise behavior.

 The study also predicted that there would be an interaction between threat and barrier on perceived self-efficacy, attitudes, and intentions to exercise such that participants in high threat and barrier-addressed message conditions would have higher perceived self-efficacy, more positive attitudes, and higher intentions to exercise than the participants in high threat and barrier not addressed message conditions. Other studies have found that there is a negative relationship between the perceived barriers to exercise and exercise participation.^28,47,48^ However, in this study, no significant interaction was found between threat and barrier on perceived self-efficacy, attitudes, and intentions to exercise. This may be because the only study addressed the barrier of time. Some of the perceived barriers to physical exercise reported in other studies included lack of motivation to exercise, lack of energy, bad weather, and school assignments.^24,27,30^ Hence, only addressing the barrier of time may not have been sufficient to increase the perceived self-efficacy of the individuals regarding exercising after reading a high-threat message. Self-efficacy might have improved despite a high-threat message if more barriers were addressed in the study. The study did find that the main effect of barriers on attitudes to exercise approached significance. This is an important finding and is in line with the HBM studies that have found the main effect of perceived barriers on attitudes, intentions, and behaviors of individuals.^18,41,43^

 The study did not find a three-way interaction of threat, efficacy, and barrier on either the attitudes or intentions to exercise. Although no three-way interaction was found, the analysis found a significant two-way interaction between efficacy and barrier on participants’ attitudes towards exercise. The result showed that the participants in the high efficacy and barrier not addressed message condition had the greatest attitudes towards exercise followed by participants in low efficacy and barrier addressed; high efficacy and barrier addressed; and low efficacy and barrier not addressed message conditions. This is an interesting finding, which suggests that when efficacy is low, addressing the barrier may be more effective in influencing the individuals’ attitudes towards recommended health behaviors than when efficacy is high. When participants are exposed to low efficacy message conditions, addressing a barrier may help to increase their perceived self-efficacy which can strengthen their attitudes towards the recommended behavior. Addressing the barrier may not be necessary when participants are exposed to a high-efficacy message. However, future studies are required to get a better understanding of how efficacy and barrier can impact the persuasiveness of a health promotion message. Also, future studies should examine the three-way interaction of threat, efficacy, and barrier in persuading individuals to follow the recommended health behaviors.

 The results did not support most of the hypotheses, maybe because the majority of participants (94.8%) reported that they had not been diagnosed with heart disease, although more than half of the participants (57%) reported that they had a close family member who has been diagnosed with heart disease. One explanation can be that people have an optimistic bias and believe that they are less likely to be affected by risk than others.^49^ The optimistic bias on self was found in a study that assessed the predictors of influenza vaccine acceptance among healthy adults.^50^ Chapman and Coups found that one of the reasons that people declined to be vaccinated against the flu was their perception that they were at low risk of getting the flu.^50^ Optimistic biases may hinder risk-reducing behaviors such that people believe they are less susceptible to disease conditions and do not take the health messages seriously.^50^ This may have led the participants in the current study to believe they are not at risk of heart disease, despite being sedentary.

###  Comparing the EPPM model vs the extended EPPM model

 While comparing the EPPM model with the Extended EPPM model proposed in this study, the study results showed that adding a barrier can strengthen the model. The addition of the barrier in the message regarding heart health increased the perceived self-efficacy of the participants in comparison to the message that did not address the barrier. The study also found a two-way interaction between barrier and efficacy such that participants in the low efficacy and barrier-addressed conditions had more positive attitudes towards exercise than the participants in the low efficacy and barrier-not addressed message conditions. The EPPM model does not include barriers, therefore addressing barriers in a low-efficacy message can help increase the attitudes of people regarding health behavior. Further research that addresses barriers in a fear appeal message regarding different health behaviors such as smoking, vaping, alcohol addiction, and others should be conducted to get a better understanding of the strength of the proposed extended EPPM model. Future studies should also test the proposed model through Structural Equation Modeling to analyze the relationship model between constructs.

 This study had several limitations. The first limitation is that the experiment was conducted online and not in a controlled experimental environment. The major drawback of an online experiment is not being able to precisely identify the participants.^51^ Several questions were asked before participants can take part in the study, but in online settings, it is difficult to ascertain participants’ demographic identity especially if the information was not provided honestly. Another issue with an online setting is the lack of environmental control. If the experiment was conducted in a controlled lab, then the researchers can make sure that there are no other distractions, and that the participants are not multitasking. The second limitation was the health message stimuli which provided narratives but lacked context. Most of the health communication studies use the message stimuli in a context of a PSA.^52-54^ However, a lack of context might have reduced the effectiveness of the message stimuli in the study. Third, this study used self-report measures. Self-reported data are subject to biases and the possibility of invalid responses. Fourth, the effect size of the barrier on participants perceived self-efficacy about exercising was small. A small effect size indicates that although statistically significant, the result has limited practical implications. Finally, this research only addressed the barrier of time for exercise and healthy diet behaviors, overlooking other barriers such as taste and cost may have been more effective in increasing the perceived self-efficacy of the participants and making the message more persuasive.

###  Future implications

 The current study should be used as an extension of EPPM that includes barriers as a component. Based on the findings, one suggestion for the health campaign messages would be to measure possible barriers that may be important before message construction. If a health campaign is using a fear appeal message, then the message should also address potential barriers to the recommended health actions to help increase the individual’s perceived self-efficacy and direct them to go through the danger control process rather than the fear control process. The findings of this study may apply to other preventive health behaviors such as getting vaccinations for COVID-19, condom use to prevent HIV/AIDS and other sexually transmitted diseases and smoking prevention.^55-58^ Addressing perceived barriers regarding preventive health behaviors may help increase the persuasiveness of the fear appeal messages. Different barriers that have been studied in health communication include language and cultural barriers, social barriers, psychological barriers, and environmental, physical, and personal barriers.^29,59-63^ Future studies should further test these barriers as appropriate in EPPM to other preventive health behaviors to design unique and effective health messages.

## Conclusion

 The findings indicated that addressing the barrier may increase an individual’s perceived self-efficacy to perform a recommended action. This is an important finding of the study because scholars have asked for fear appeal studies to identify and address barriers to increasing self-efficacy.^26,27^ This study confirms the assumption of the previous studies that addressing barriers can be one way of increasing individuals’ confidence about their ability to follow the recommended actions in a health message. This finding adds a meaningful variable to EPPM that needs to be studied further to gain a better understanding of how addressing barriers may influence individuals’ self-efficacy regarding other health behaviors.

## Author Contributions

**Conceptualization:** Rashmi Thapaliya, Glenn Leshner.

**Data curation: **Rashmi Thapaliya, Amir Bhochhibhoya.

**Formal analysis:** Rashmi Thapaliya, Glenn Leshner, Amir Bhochhibhoya, Pragya Sharma-Ghimire.

**Investigation:** Rashmi Thapaliya.

**Project administration:** Amir Bhochhibhoya, Rashmi Thapaliya.

**Supervision:** Glenn Leshner.

**Writing-original draft:** Rashmi Thapaliya, Amir Bhochhibhoya, Pragya Sharma-Ghimire.

**Writing-review and editing**: Amir Bhochhibhoya, Rashmi Thapaliya.

## Funding

 This research received no specific grant.

## Ethical Approval

 This study was approved by the Institutional Review Board at the University of Oklahoma (Protocol number 9367).

## Competing Interests

 None.

## References

[1] Heron M (2019). Deaths: leading causes for 2017. Natl Vital Stat Rep.

[2] Benjamin EJ, Muntner P, Alonso A, Bittencourt MS, Callaway CW, Carson AP (2019). Heart disease and stroke statistics-2019 update: a report from the American Heart Association. Circulation.

[3] Center for Disease Control and Prevention (CDC). Heart Disease. Available from: https://www.cdc.gov/heartdisease/prevention.htm. Accessed August 8, 2022.

[4] Fryar CD, Chen TC, Li X. Prevalence of uncontrolled risk factors for cardiovascular disease: United States, 1999-2010. NCHS Data Brief. 2012(103):1-8. 23101933

[5] Mellett LH, Bousquet G (2013). Cardiology patient page. Heart-healthy exercise. Circulation.

[6] National Center for Health Statistics. Healthy People 2010. Available from: https://www.cdc.gov/nchs/healthy_people/hp2010.htm. Accessed August 8, 2022.

[7] Michie S, Prestwich A (2010). Are interventions theory-based? Development of a theory coding scheme. Health Psychol.

[8] Leshner G, Bolls P, Wise K (2011). Motivated processing of fear appeal and disgust images in televised anti-tobacco ads. J Media Psychol.

[9] Leventhal H. Findings and theory in the study of fear communications. In: Berkowitz L, ed. Advances in Experimental Social Psychology. Vol 5. Academic Press; 1970. p. 119-86. 10.1016/s0065-2601(08)60091-x

[10] Witte K (1992). Putting the fear back into fear appeals: the extended parallel process model. Commun Monogr.

[11] Witte K (1994). Fear control and danger control: a test of the extended parallel process model (EPPM). Commun Monogr.

[12] Champion VL, Skinner CS. The health belief model. In: Glanz K, Rimer BK, Viswanath K, eds. Health Behavior and Health Education: Theory, Research, and Practice. Jossey-Bass; 2008. p. 45-65.

[13] Noar SM (2005). A health educator’s guide to theories of health behavior. Int Q Community Health Educ.

[14] Noar SM, Zimmerman RS (2005). Health Behavior Theory and cumulative knowledge regarding health behaviors: are we moving in the right direction?. Health Educ Res.

[15] Cappella JN (2006). Integrating message effects and behavior change theories: organizing comments and unanswered questions. J Commun.

[16] Austin LT, Ahmad F, McNally MJ, Stewart DE (2002). Breast and cervical cancer screening in Hispanic women: a literature review using the health belief model. Womens Health Issues.

[17] Jung T, Brann M (2014). Analyzing the extended parallel process model and health belief model constructs in texting while driving: news coverage in leading US news media outlets. Int J Health Promot Educ.

[18] Carpenter CJ (2010). A meta-analysis of the effectiveness of health belief model variables in predicting behavior. Health Commun.

[19] Janz NK, Becker MH (1984). The health belief model: a decade later. Health Educ Q.

[20] Carcioppolo N. Assessing the Utility of Integrating Perceived Barrier and Response Cost Measures into the Extended Parallel Process Model [dissertation]. Ann Arbor: State University of New York at Buffalo; 2008.

[21] So J (2013). A further extension of the extended parallel process model (E-EPPM): implications of cognitive appraisal theory of emotion and dispositional coping style. Health Commun.

[22] Hong H (2011). An extension of the extended parallel process model (EPPM) in television health news: the influence of health consciousness on individual message processing and acceptance. Health Commun.

[23] Allison KR, Dwyer JJ, Makin S (1999). Perceived barriers to physical activity among high school students. Prev Med.

[24] Booth ML, Bauman A, Owen N, Gore CJ (1997). Physical activity preferences, preferred sources of assistance, and perceived barriers to increased activity among physically inactive Australians. Prev Med.

[25] Kearney JM, McElhone S (1999). Perceived barriers in trying to eat healthier--results of a pan-EU consumer attitudinal survey. Br J Nutr.

[26] Hosseini H, Moradi R, Kazemi A, Shahshahani MS (2017). Determinants of physical activity in middle-aged woman in Isfahan using the health belief model. J Educ Health Promot.

[27] Kasser SL, Kosma M (2012). Health beliefs and physical activity behavior in adults with multiple sclerosis. Disabil Health J.

[28] Mo PK, Chong ES, Mak WW, Wong SY, Lau JT (2016). Physical activity in people with mental illness in Hong Kong: application of the health belief model. J Sport Exerc Psychol.

[29] Dwyer JJ, Allison KR, Goldenberg ER, Fein AJ, Yoshida KK, Boutilier MA (2006). Adolescent girls’ perceived barriers to participation in physical activity. Adolescence.

[30] Pratt CA, Ha L, Levine SR, Pratt CB (2003). Stroke knowledge and barriers to stroke prevention among African Americans: implications for health communication. J Health Commun.

[31] Tergerson JL, King KA (2002). Do perceived cues, benefits, and barriers to physical activity differ between male and female adolescents?. J Sch Health.

[32] Kees J, Berry C, Burton S, Sheehan K (2017). An analysis of data quality: professional panels, student subject pools, and Amazon’s Mechanical Turk. J Advert.

[33] Murphy ST, Frank LB, Chatterjee JS, Baezconde-Garbanati L (2013). Narrative versus non-narrative: the role of identification, transportation and emotion in reducing health disparities. J Commun.

[34] Flowers A. The Most Common Unisex Names in America: Is Yours One of Them? Available from: https://fivethirtyeight.com/features/there-are-922-unisex-names-in-america-is-yours-one-of-them/#:~:text = 9%3A16%20AM-,The%20Most%20Common%20Unisex%20Names%20In,Is%20Yours%20One%20Of%20Them%3F&text = Casey%2C%20Riley%2C%20Jessie%20and%20Jackie. Accessed August 8, 2022.

[35] Richards JA, Johnson MP (2014). A case for theoretical integration: combining constructs from the theory of planned behavior and the extended parallel process model to predict exercise intentions. SAGE Open.

[36] Carcioppolo N, Jensen JD, Wilson SR, Collins WB, Carrion M, Linnemeier G (2013). Examining HPV threat-to-efficacy ratios in the extended parallel process model. Health Commun.

[37] McKay DL, Berkowitz JM, Blumberg JB, Goldberg JP (2004). Communicating cardiovascular disease risk due to elevated homocysteine levels: using the EPPM to develop print materials. Health Educ Behav.

[38] Slonim AB, Roberto AJ, Downing CR, Adams IF, Fasano NJ, Davis-Satterla L (2005). Adolescents’ knowledge, beliefs, and behaviors regarding hepatitis B: insights and implications for programs targeting vaccine-preventable diseases. J Adolesc Health.

[39] Witte K (1997). Preventing teen pregnancy through persuasive communications: realities, myths, and the hard-fact truths. J Community Health.

[40] Witte K, Girma B, Girgre A (2002). Addressing underlying mechanisms to HIV/AIDS preventive behaviors in Ethiopia. Int Q Community Health Educ.

[41] Becker MH, Maiman LA, Kirscht JP, Haefner DP, Drachman RH (1977). The health belief model and prediction of dietary compliance: a field experiment. J Health Soc Behav.

[42] Blue CL, Valley JM (2002). Predictors of influenza vaccine. Acceptance among healthy adult workers. AAOHN J.

[43] Champion VL (1990). Breast self-examination in women 35 and older: a prospective study. J Behav Med.

[44] Hyman RB, Baker S, Ephraim R, Moadel A, Philip J (1994). Health belief model variables as predictors of screening mammography utilization. J Behav Med.

[45] Kühner MK, Raetzke PB (1989). The effect of health beliefs on the compliance of periodontal patients with oral hygiene instructions. J Periodontol.

[46] McArthur LH, Riggs A, Uribe F, Spaulding TJ (2018). Health belief model offers opportunities for designing weight management interventions for college students. J Nutr Educ Behav.

[47] Al-Ali N, Haddad LG (2004). The effect of the health belief model in explaining exercise participation among Jordanian myocardial infarction patients. J Transcult Nurs.

[48] Moore JB, Jilcott SB, Shores KA, Evenson KR, Brownson RC, Novick LF (2010). A qualitative examination of perceived barriers and facilitators of physical activity for urban and rural youth. Health Educ Res.

[49] Weinstein ND (1989). Optimistic biases about personal risks. Science.

[50] Chapman GB, Coups EJ (1999). Predictors of influenza vaccine acceptance among healthy adults. Prev Med.

[51] Horton JJ, Rand DG, Zeckhauser RJ (2011). The online laboratory: conducting experiments in a real labor market. Exp Econ.

[52] Fishbein M, Hall-Jamieson K, Zimmer E, von Haeften I, Nabi R (2002). Avoiding the boomerang: testing the relative effectiveness of antidrug public service announcements before a national campaign. Am J Public Health.

[53] Phua J (2016). The effects of similarity, parasocial identification, and source credibility in obesity public service announcements on diet and exercise self-efficacy. J Health Psychol.

[54] Dillard JP, Peck E (2000). Affect and persuasion: emotional responses to public service announcements. Commun Res.

[55] Sarkar NN (2008). Barriers to condom use. Eur J Contracept Reprod Health Care.

[56] Gharlipour Z, Hazavehei SM, Moeini B, Nazari M, Moghim Beigi A, Tavassoli E (2015). The effect of preventive educational program in cigarette smoking: extended parallel process model. J Educ Health Promot.

[57] Wong NC, Cappella JN (2009). Antismoking threat and efficacy appeals: effects on smoking cessation intentions for smokers with low and high readiness to quit. J Appl Commun Res.

[58] Thrasher JF, Swayampakala K, Borland R, Nagelhout G, Yong HH, Hammond D (2016). Influences of self-efficacy, response efficacy, and reactance on responses to cigarette health warnings: a longitudinal study of adult smokers in Australia and Canada. Health Commun.

[59] Robinson M, Gilmartin J (2002). Barriers to communication between health practitioners and service users who are not fluent in English. Nurse Educ Today.

[60] Uba L (1992). Cultural barriers to health care for southeast Asian refugees. Public Health Rep.

[61] O’Dea J A (2003). Why do kids eat healthful food? Perceived benefits of and barriers to healthful eating and physical activity among children and adolescents. J Am Diet Assoc.

[62] Chen MF, Wang RH, Schneider JK, Tsai CT, Jiang DD, Hung MN (2011). Using the health belief model to understand caregiver factors influencing childhood influenza vaccinations. J Community Health Nurs.

[63] Frisby CM (2002). Messages of hope: health communication strategies that address barriers preventing black women from screening for breast cancer. J Black Stud.

